# Long‐term rehabilitation with interferential current stimulation for persistent dysphagia in anti‐N‐methyl‐d‐aspartate receptor encephalitis: A case report

**DOI:** 10.1002/ccr3.9307

**Published:** 2024-08-12

**Authors:** Takahiro Sugahara, Shinsuke Nagami, Ryota Sato, Naohiko Ishizaki, Ichiro Fujishima, Fumihito Aikawa

**Affiliations:** ^1^ Department of Rehabilitation Aikawa Clinic, Medical Corporation Yamaguchi Japan; ^2^ Department of Communication Disorders, School of Rehabilitation Sciences Health Sciences University of Hokkaido Tobetsu Hokkaido Japan; ^3^ Department of Neurology and Clinical Neuroscience Yamaguchi University Graduate School of Medicine Yamaguchi Japan; ^4^ Department of Rehabilitation Medicine Hamamatsu City Rehabilitation Hospital Shizuoka Japan; ^5^ Aikawa Clinic, Medical Corporation Yamaguchi Japan

**Keywords:** dysphagia, interferential current, N‐methyl‐d‐aspartate, resistance excise

## Abstract

Anti‐N‐methyl‐d‐aspartate receptor encephalitis is an autoimmune disorder characterized by various neurological symptoms with a relatively favorable prognosis. We present a case of prolonged dysphagia successfully managed with outpatient rehabilitation, including interferential current stimulation and resistance exercises. Significant improvement was observed, highlighting the efficacy of combined treatment in overcoming chronic dysphagia.

## 1 INTRODUCTION

Anti‐N‐methyl‐d‐aspartate (NMDA) receptor encephalitis, an autoimmune disease, leads to autoantibody production against NMDA‐type glutamate receptors.^1^ Recently, an increasing number of cases of anti‐NMDA receptor encephalitis have been reported, including those without tumors.^2^, ^3^, ^4^ Clinical features include five stages: prodromal, psychotic, unresponsive, involuntary movement, and slow recovery.^5^ Approximately 81% of patients experience favorable outcomes following a 24‐month follow‐up, while the rest experience relapses or succumb to the disease.^4^

In our clinical practice, we experienced a case of anti‐NMDA receptor encephalitis with severe chronic dysphagia that necessitated prolonged outpatient rehabilitation. Detailed reports on long‐standing dysphagia following a negative cerebrospinal fluid test for this condition are scarce. To the best of our knowledge, the literature on this topic includes the following: a report on dysphagia during the unresponsive stage,^6^ a case report of pseudobulbar palsy dysphagia without abnormal findings on magnetic resonance imaging (MRI),^7^ and an observational study in which dysphagia occurred in 15% of 167 children and adolescents with anti‐NMDA receptor encephalitis.^8^ Thus, persistent dysphagia in these cases remains unclear, whether from sustained neurological impairment or muscle weakness. Despite improvements in dysarthria and orofacial dyskinesia over time, dysphagia often remains persistent. Furthermore, reports on long‐term, continuous interventions for feeding and swallowing rehabilitation are notably lacking.

Interventions include transcutaneous electrical sensory stimulation therapy and resistance exercises. Our management strategy combines both methods. The neuroelectrical approach focuses on sensory inputs associated with triggering swallowing reflexes. We employed interferential current (IFC) stimulation therapy to address delayed swallowing reflexes, aiming to stimulate swallowing. This method has been deemed safe by our group and has the potential to induce neurological improvement in feeding and swallowing disorders.^9^ This report presents a case of anti‐NMDA receptor encephalitis with feeding and swallowing disorders and provides details of the outpatient rehabilitation process.

## 2 CASE HISTORY/EXAMINATION

**FIGURE 1:**
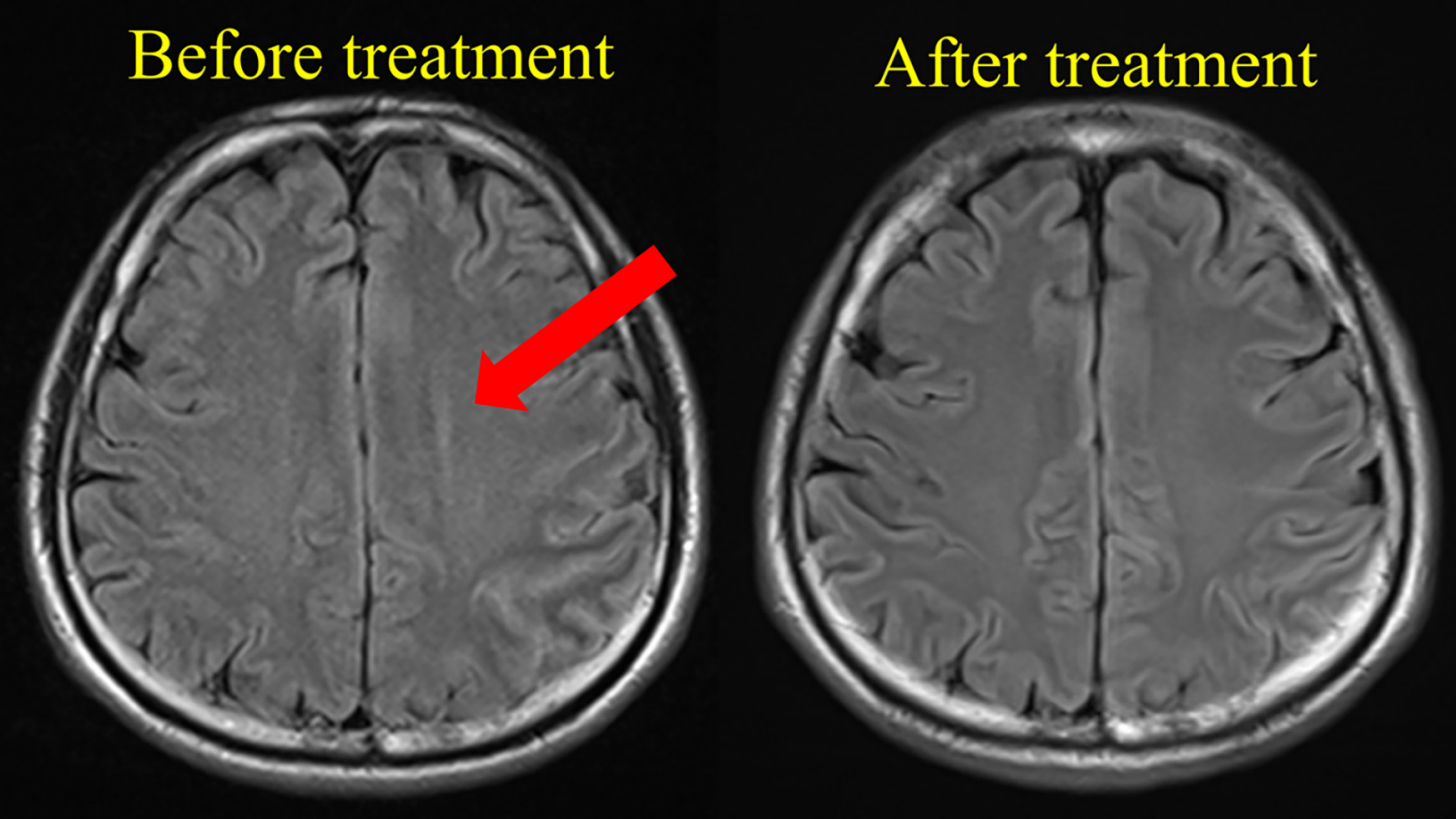
Head magnetic resonance imaging (MRI). MRI images before and after treatment were compared. Before treatment, a high fluid‐attenuated inversion recovery signal intensity was observed in the left parietal cortex. No lesion was observed in the right cerebrum or brainstem.

A 48‐year‐old man was admitted to an acute care hospital with primary symptoms of right‐hand dyskinesia and dysarthria in July 2015. Cerebrospinal fluid examination demonstrated an increased cell count, mildly elevated protein levels, and anti‐NMDA receptor antibodies. The cell count was 102/dL, protein level was 54.7 mg/dL, and glucose level was 59.0 mg/dL. Fluid‐attenuated inversion recovery MRI of the head showed a high‐signal area in the left parietal cortex (Figure 1). After admission, the patient's condition deteriorated, and impaired consciousness, aspiration pneumonia, delusions, and abnormal behavior developed. Based on these findings, the patient was diagnosed with anti‐NMDA receptor encephalitis. During the hospitalization period, the patient experienced difficulty with oral intake and swallowing rehabilitation due to impaired consciousness. Endotracheal intubation was not required. Tracheostomy was performed to prevent aspiration, not due to respiratory failure. Mechanical ventilation was used for only 1 day (from the day of the tracheostomy surgery to the following day). The patient received nutrition through an 8‐Fr nasogastric tube.

**FIGURE 2:**
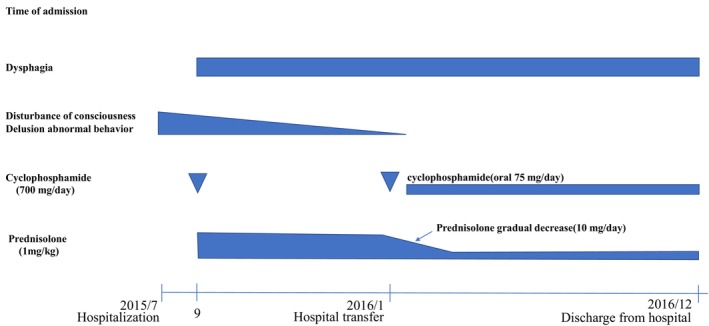
Clinical course of the previous medical institution (acute care hospital–convalescent hospital). The course of the disease before visiting our hospital. Fifteen months have passed since the patient developed anti‐N‐methyl‐d‐aspartate (NMDA) receptor encephalitis. Immunotherapy was initiated after the patient was diagnosed with anti‐NMDA receptor encephalitis. Dysphagia persisted after NMDA receptor antibodies became negative.

In September 2015, immunotherapy was administered with steroids (prednisolone 1 mg/kg for 8 weeks) and cyclophosphamide pulse therapy (700 mg/day). Subsequently, the patient's neurological symptoms, such as impaired consciousness, improved, and brain and spinal findings normalized. With continued immunotherapy, the patient was transferred to a convalescent hospital in January 2016. The prednisolone dosage was gradually tapered to 10 mg/day, and cyclophosphamide was transitioned to oral administration (75 mg/day) after the second course of pulse therapy (Figure 2). Cyclophosphamide was discontinued once the NMDA receptor antibodies turned negative.

After being transferred to the convalescent hospital, the patient continued to exhibit dysphagia. Thus, a videoendoscopic examination of swallowing (VE) was performed in February 2016. An initial VE evaluation revealed a substantial accumulation of saliva in the laryngeal vestibule and pyriform sinus. Moreover, the patient exhibited poor swallowing reflexes, impaired laryngeal elevation and pharyngeal contraction, and lack of epiglottic movement. Although cough and glottal closure reflexes were observed, the patient remained unaware of pharyngeal residuals. The swallowing endoscopic examination was evaluated using the Hyodo score. The results were as follows: salivary pooling degree G2, glottal closure reflex G0, swallowing reflex initiation assessed G3, pharyngeal clearance G3.^10^ During the course of the disease, he demonstrated self‐harm and harmful behaviors toward others, accompanied by anger and restlessness. Consequently, regarding rehabilitation, we implemented indirect training that focused primarily on the delayed swallowing reflex and laryngeal elevation impairment. This included oral care, ice massage of the throat, Mendelsohn's maneuver, and Shaker exercises. The patient's mental state stabilized. Although abnormal behaviors ceased, dysphagia did not improve. We observed no significant changes in the Hyodo score at the time of discharge from the recovery phase hospital. The previous physician (at the recovery phase hospital) deemed the patient unsuitable for oral intake, and the patient underwent percutaneous endoscopic gastrostomy (PEG) installation. Following this, no significant weight loss was observed, which was likely due to careful weight management. The patient was fed in the morning and evening (1500–2000 kcal/day), with the amount adjusted based on changes in weight.

Driven by his desire to reintegrate into society, the patient was discharged from the convalescent hospital in January 2017 with persistent dysphagia. Despite long‐term difficulties in oral intake and the mental and physical burdens of PEG, the patient, undeterred, sought continued rehabilitation for oral intake and began outpatient swallowing rehabilitation at our facility.

## 3 METHODS

### 3.1 Investigation

The patient was self‐sufficient, except for the need for tube feeding. His height was 166 cm, weight was 54 kg, blood pressure was 107/81 mmHg, oxygen saturation (SpO_2_) was 98%, body mass index was 19.5 kg/m^2^, and functional independence measure was 125/126; no abnormalities were observed upon general physical examination. Additionally, we did not find any irregularities in the patient's motor, sensory, cerebellar, or autonomic nervous systems. The blood test results revealed no abnormalities, with values recorded as follows: white blood cell count 5.6/10^3^/μL, total protein 6.7/dL, albumin 4.1/dL, hemoglobin 13.6 g/dL, and C‐reactive protein 0.11 mg/dL. The repetitive saliva swallowing test (RSST) was difficult to perform because the swallowing reflex could not be elicited.^11^ Regarding the modified water swallowing test, he was categorized as 3a (wet hoarseness with no swallowing).^12^ In terms of the Food Intake Level Scale (FILS), he was at level 3 (swallowing training with a small amount of food).^13^ Regarding the Eating Assessment Tool (EAT‐10), he scored 40/40, with three or more points indicating suspected dysphagia.^14^

**FIGURE 3:**
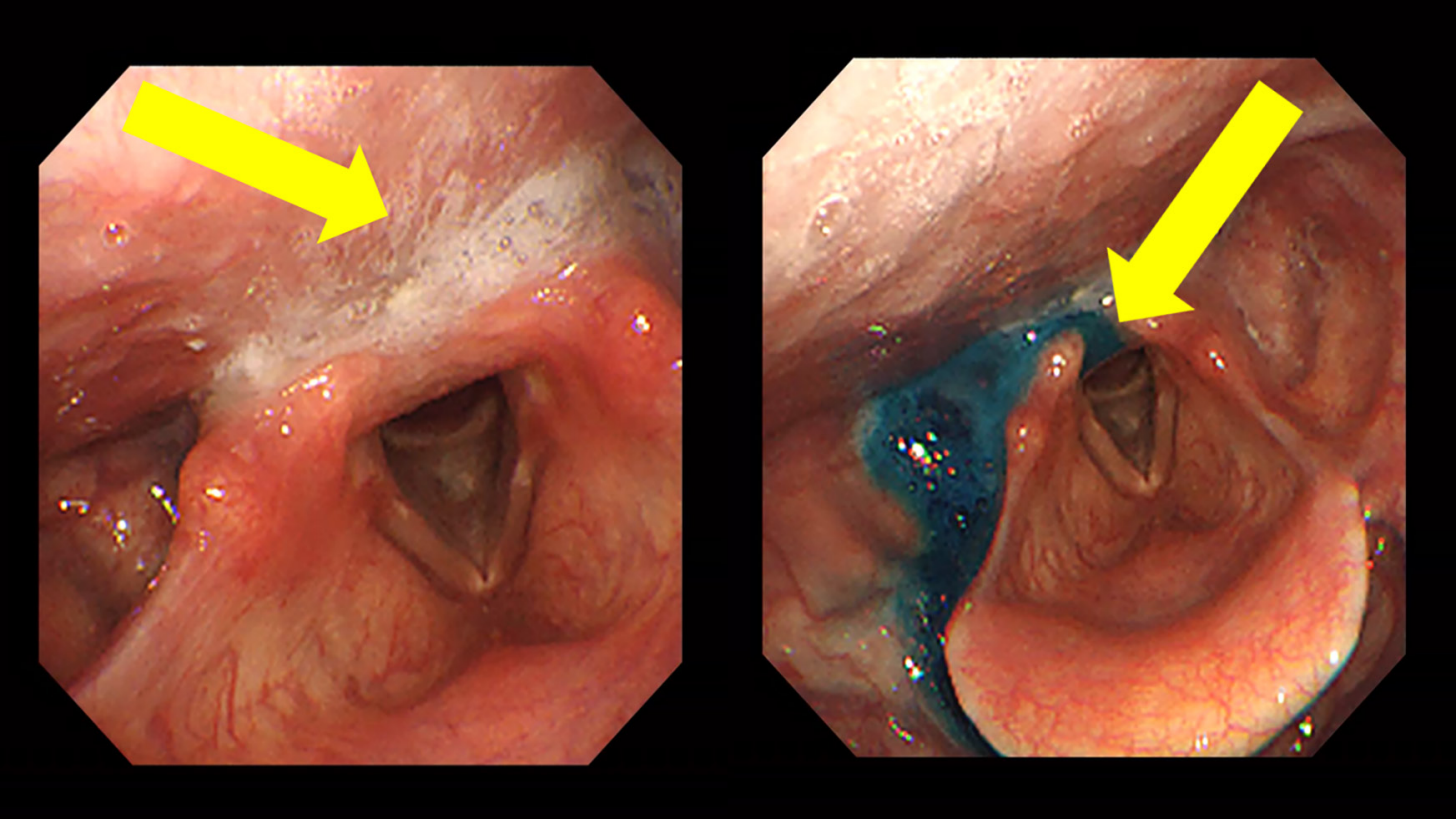
Videoendoscopic examination of swallowing. Swallowing evaluation upon patient presentation to the outpatient setting. (Left) Saliva retention observed at rest. (Right) Findings of colored water penetration into the larynx. Whiteout was not observed, indicating inadequate closure of the larynx and decreased pharyngeal contractile function. The patient swallowed multiple times, but laryngeal intrusion was observed.

VE showed saliva retention at rest (Figure 3). Upon the intake of 3 mL of colored water, a delay in swallowing reflex induction, poor laryngeal elevation, and poor pharyngeal constriction with no whiteout were noted. Cough and glottal closure reflexes were observed, but the residuals in the epiglottis valley and pisiform fossa were not removed by multiple swallowing attempts, leading to laryngeal intrusion.

During the videofluoroscopic examination of swallowing (VF) study, the patient swallowed 3 g of barium jelly while in the seated position. The study revealed a delayed swallowing reflex of 2.3 s, poor pharyngeal contraction, and inadequate laryngeal elevation. Reflex induction time was assessed based on the laryngeal elevation delay time (LEDT), with a cutoff value set at 0.35 s, according to the findings of a previous study, which established normal values for reflex induction time (0.35 s).^15^ The patient's reflex induction time was significantly prolonged compared with the normal cutoff of 0.35 s, indicating a delayed swallowing reflex. There was no lateral difference in residue, and the bolus passed through both sides; however, residues were noted in the vallecula and pyriform sinuses. Considering the information obtained from the previous physician and the risk of aspiration pneumonia, we decided to conclude the examination with only indirect training once laryngeal penetration was observed. The Penetration‐Aspiration Scale (PAS) score was 5 (material enters the airway, contacts the vocal folds, and is not ejected from the airway).^16^ Surgical procedures such as laryngeal elevation and cricopharyngeal myotomy were proposed, but the patient preferred conservative treatment and expressed his desire to continue with outpatient rehabilitation.

### 3.2 Treatment (dysphagia rehabilitation)

A structured training plan was implemented for dysphagia rehabilitation. First, respiratory rehabilitation was performed, focusing on the vocal cords. Respiratory therapy with hard blowing was performed to address respiratory muscle weakness. The patient performed continuous exhalation activities as if a tissue paper was fluttering while taking. Hard blowing was performed since it could be done within 5 min. Pushing and pulling exercises were performed to fortify the glottal defense mechanism (2 sets of 5 repetitions).^17^ Shaker exercises were performed to treat poor laryngeal elevation, with the intensity and frequency adjusted to match the patient's physical capacity. Shaker exercises comprised maintaining head flexion for 1 min in the supine position (3 sets) with 1 min rest after each set and performing repetitive head flexion and extension movements 30 times, 3 times/day.^18^ Furthermore, the following exercises were performed: Mendelsohn's maneuver, consisting of maintaining the elevated laryngeal position (5 sets) (if difficult, manual assistance can be used), with a mirror being used to check laryngeal movement^19^; mouth‐opening exercises, consisting of maintaining maximum mouth opening for 10 s with 10 s rest between sets (5 sets of 2 repetitions)^20^; and tongue elevation exercises, consisting of pressing the tongue against the hard palate for 3–5 s (10 times), 2–3 sets/day, with the load being set to 60%–80% of maximum muscle strength.^21^ Additionally, the patient was instructed to perform home exercises daily. A mirror and video were used to follow the same protocol (Table 1).

**TABLE 1:** IFC stimulation therapy and resistance exercise protocol.

*Shaker exercises* Maintaining head flexion for 1 min in the supine position (3 sets) with 1 min rest after each set, and performing repetitive head flexion and extension movements 30 times, 3 times/day
*Mendelsohn's maneuver* Consisting of maintaining the elevated laryngeal position (5 sets) (if difficult, manual assistance can be used) with a mirror being used to check laryngeal movement
*Mouth‐opening exercises* Consisting of maintaining maximum mouth opening for 10 s with 10 s rest between sets (5 sets of 2 repetitions)
*Tongue elevation exercises* Consisting of pressing the tongue against the hard palate for 3–5 s (10 times) 2–3 sets/day with the load being set to 60%–80% of maximum muscle strength
*IFC stimulation therapy* Stimulation electrodes were position on the skin of the bilateral anterior neck 20 min (2.5–3 mA) of electric current once or twice a week After 4 months, conducted once every day daily at home

*Note*: A mirror and video were used to follow the same protocol.

Next, dysphagia training was performed to treat sensory impairment. We employed ice massage of the throat and IFC stimulation using a Gentle Stim® (FoodCare) stimulator with a beat frequency of 50 Hz and a carrier frequency of 2000 Hz to expedite the swallowing reflex.^22^ Stimulation electrodes were positioned on the skin of the bilateral anterior neck.^9^ The duration of the stimulation sessions was 20 min (2.5–3 mA) once or twice a week during outpatient rehabilitation from the initial intervention to 4 months. The patient desired to perform IFC stimulation daily at home. Therefore, we authorized the daily conduct of IFC stimulation at home. He should follow the same protocol used in clinic visits. To ensure home use, he was given thorough instruction on how to use IFC stimulation under the supervision of a medical doctor and speech therapist. Thus, 4 months later, the patient was able to perform IFC stimulation daily at home.

## 4 CONCLUSIONS AND RESULTS

### 4.1 Conclusion

Herein, the patient experienced long‐term dysphagia. However, IFC stimulation and resistance training ameliorated swallowing dysfunction. Although anti‐NMDA receptor encephalitis may induce long‐term dysphagia, we acknowledge the limitations of generalizing this point due to the insufficient number of cases and the possibility that NMDA receptor encephalitis may not be the sole cause of dysphagia. Consequently, we cannot yet generalize our findings, although we eagerly anticipate further accumulation of cases that could support such generalization in the future. Continued swallowing rehabilitation with immunotherapy may improve dysphagia.

### 4.2 Results (clinical course)

**FIGURE 4:**
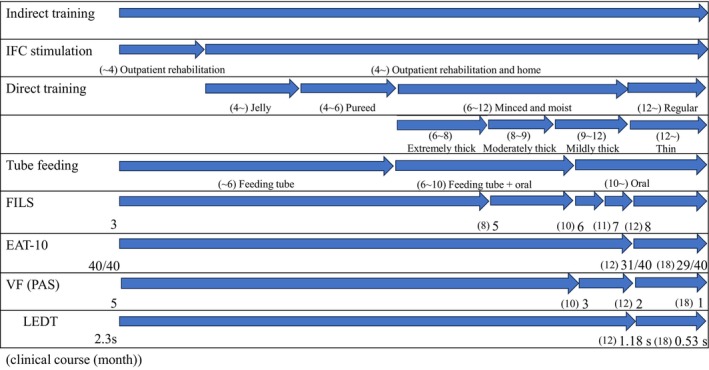
Clinical course of the patient's visit to our clinic. Percutaneous sensory electrical stimulation was started in parallel with basic non‐food swallowing training (indirect training). Training in the use of food (direct training) was added as the patient's condition improved. Eventually, the patient improved to the point of eating three meals orally without the feeding tube.

The EAT‐10 score improved from 40/40 to 29/40. The FILS level improved from level 3 (swallowing training with a small amount of food) to level 8 (the patient eats three meals by excluding food that is particularly difficult to swallow). The PAS score improved from 5 (material enters the airway, contacts the vocal folds, and is not ejected from the airway) to 1 (material does not enter the airway). The LEDT shortened from 2.3 to 0.53 s. Food consistency improved from jelly to regular.^23^ Fluid consistency improved from extremely thick to thin. Tube feeding improved from feeding tube (PEG) to oral feeding (Figure 4). Although no lateral difference in residue was observed during the VF examination, the patient used compensatory techniques due to anxiety and fear. Upon reassurance and repeated explanations, the patient stopped using these techniques.

The swallowing disorder was completely cured. However, the patient continued to visit the outpatient clinic twice a month to maintain swallowing function and to continue resistance exercises upon his request. Additionally, he continued IFC stimulation therapy at home once every 1–2 days.

## 5 DISCUSSION

We presented a case of anti‐NMDA receptor encephalitis with dysphagia associated with a severe delay in swallowing reflexes and weakness of swallowing‐related muscles. Although no conspicuous neurological impairment of the speech organs was observed, velopharyngeal incompetence and poor laryngeal elevation were present. Immunotherapy and swallowing rehabilitation improved dysphagia gradually. The long‐term amelioration is consistent with the known characteristics of anti‐NMDA receptor encephalitis, a condition that typically improves over several years.^4^

Herein, we investigated the effects of IFC stimulation therapy on delayed swallowing reflexes and muscle weakness in a patient with dysphagia. IFC stimulation of the superior laryngeal nerve has been reported to improve sensory disturbances and induce swallowing reflexes. Umezaki et al. demonstrated that this stimulation activates swallowing‐related neurons.^24^ Yoshimatsu et al. reported improvements in EAT‐10 and RSST scores with IFC stimulation.^25^ The long‐term application of IFC in this case resulted in decreased pharyngeal residue, shortened reflex time, and increased oral intake. Herein, the MRI findings did not correlate directly with dysphagia. Although some cases of anti‐NMDA receptor encephalitis show abnormal MRI findings, many do not, suggesting that NMDA receptor dysfunction might extend beyond the detectable brain regions.^1^, ^4^, ^26^ This dysfunction could be the underlying cause of severe delay in swallowing reflex induction and weakness.

In this case, dysphagia was not due to sarcopenia, as indicated by normal blood tests and the absence of muscle weakness in the limbs and trunk. Furthermore, the patient did not meet the diagnostic criteria for sarcopenic dysphagia.^27^ Considering the patient's self‐managed nutritional status and independence in ADL, the dysphagia might result from hospital‐associated dysphagia^28^ or neuropathy due to anti‐NMDA receptor encephalitis. The patient's weight, monitored by his prior physician, did not present excessive fluctuations compared with his weight during his prior hospitalization, and blood tests revealed no abnormalities. However, the patient exhibited pharyngeal contractile dysfunction and poor laryngeal elevation. Consequently, we applied progressive resistance exercises to the speech and articulation organs. Recently, resistance training, particularly progressive strength training, has proven effective. Dehaghani et al. implemented resistance training on healthy older subjects for 4 weeks and reported improved swallowing function.^29^ Robbins et al. implemented resistance training on patients with stroke‐induced dysphagia for 8 weeks and reported improved swallowing pressure.^21^

Despite these findings, this study has several limitations. Unfortunately, we had only limited information on the patient's history from the previous hospital. The potential for a strong neurological impact or the influence of immunotherapy on neurological improvement due to muscle weakness should be considered. Moreover, we did not confirm whether IFC stimulation therapy had an immediate effect, and we cannot definitively state whether transcutaneous electrical sensory stimulation therapy contributed to neurological improvement or whether resistance training was effective.

## AUTHOR CONTRIBUTIONS

**Takahiro Sugahara:** Conceptualization; supervision; writing – original draft; writing – review and editing. **Shinsuke Nagami:** Conceptualization; writing – original draft; writing – review and editing. **Ryota Sato:** Data curation; writing – review and editing. **Naohiko Ishizaki:** Conceptualization; writing – review and editing. **Ichiro Fujishima:** Conceptualization; supervision; writing – review and editing. **Fumihito Aikawa:** Supervision; writing – review and editing.

## CONFLICT OF INTEREST STATEMENT

This study was conducted with financial support from Food Care Co., Ltd. The funding source had no involvement in the study design, data collection, analysis, interpretation, or manuscript preparation. The authors maintained full independence in the research process and had complete editorial freedom in reporting the results.

## CONSENT

Written informed consent was obtained from the patient in accordance with the journal's patient consent policy.

## Data Availability

Data sharing not applicable—no new data generated. Data sharing is not applicable to this article as no new data were created or analyzed in this study.
